# Analgesic efficacy of posterior and anterior psoas compartment block: Lumbar plexus versus three -in-one nerve block after lower limb orthopedic surgery under spinal anesthesia: A prospective cohort study

**DOI:** 10.1016/j.amsu.2021.103160

**Published:** 2021-12-06

**Authors:** Habtu Adane Aytolign, Abraham Tarekegn Mersha, Yonas Admasu Ferede

**Affiliations:** Department of Anaesthesia and Critical Care, College of Medicine and Health Science, University of Gondar, Gondar, Ethiopia

**Keywords:** *Lumbar plexus*, *Three-in-one*, *Nerve block*, *Orthopedic surgery*, *Analgesia*, *Spinal anesthesia*

## Abstract

**Introduction:**

Postoperative pain is the most common complaint in patients who underwent orthopedic surgery. Regarding with the severity of pain, orthopedic patients suffered more than non-orthopedic patients in the immediate post-operative period. Therefore, pain management is crucial for better patient outcome. Lumbar plexus (LB) and three -in-one (3IN1) nerve blocks have been routinely practiced as pain management techniques in the study area but the analgesic efficacy was not studied yet. Thus, this study was aimed to compare the analgesic efficacy of the LBP versus 3IN1B as postoperative pain management after thigh orthopedic surgery under spinal anesthesia.

**Method:**

An institutional-based prospective cohort study was conducted from October 10, 2020 to March 30, 2021 at the University comprehensive specialized hospital. Non-probability convenient sampling was used to select participants in both groups. The time to first analgesic request, severity of pain and total analgesia consumption within the first postoperative 24 h were measured.

**Result:**

The mean and standard deviation to seek the first analgesia request time was 11. 55 ± 2. 82hr and 13. 35 ± 2. 58hr (p- 0.07) in patients who received LPB and 3IN1B respectively. Pain severity at rest and on movement was also comparable. The total tramadol consumption was 67. 65 ± 27. 20 mg and 70. 59 ± 37. 19 mg (p- 0.71), while total Diclofenac consumption was 63. 23 ± 45. 74 mg and 44. 88 ± 34. 72 mg (p-0.07) in LPB and 3IN1B groups respectively.

**Conclusion:**

The study showed that there was no significant difference in the time to first analgesia request, postoperative pain, both at rest and movement and total analgesic consumption, between the LPB and 3IN1B.

## Highlights

• Orthopedic patients suffered more than non-orthopedic patients in the immediate post-operative period.
• Nerve blocks provide effective analgesic options.
• There are controversies comparing the efficacy of lumbar plexus and three in one femoral nerve block.
• Lumbar plexus and three in one nerve blocks have comparable time to seek 1st analgesia.
• Lumbar plexus and three in one nerve blocks have comparable pain severity and comparable analgesic consumption.

## 1 Introduction

Regarding the severity of pain, orthopedic patients suffered more than non-orthopedic patients in the immediate post-operative period [1]. Therefore, Pain management is crucial after orthopedic surgery [2]. Furthermore, Postoperative pain is a *common* complaint in patients who underwent lower limb orthopedic surgery [3,4]. Suboptimal postoperative pain management *has also been associated* with *the development* of chronic pain, economic burden, delay recovery, immobilization leading to deep venous thrombosis, pulmonary embolism, myocardial ischemia and stroke which can further delay in hospital stay [5].

There are many options of lower *limb pain management, specifically* the thigh orthopedic pain *management, including* epidural, posterior and anterior lumbar plexus block and multimodal analgesia [[6], [7], [8]]. But optimal pain management is still controversial. However, many scholars suggested that psoas compartment nerve blocks provide effective analgesic options as multimodal approaches by decreasing opioid and others analgesic consumption and related side effects [9,10].

The LPB and 3IN1B were first introduced by Winnie [11]. The LPB (from 1st lumbar nerve to 4th lumbar nerve) is used to block the iliohypogastric nerve, Ilioinguinal nerve, genitofemoral nerve, femoral nerve, obturator nerve, and lateral cutaneous nerve of the thigh [12]. Whereas, The 3IN1B is also the technique to block the femoral nerve, the lateral cutaneous nerve of the thigh, and obturator nerves of the medial thigh in a single needle insertion point [13].

There are still different controversies comparing the efficacy of single shot LPB and 3IN1B for lower limb orthopedic surgery [[14], [15], [16], [17], [18]]. Furthermore, those blocks *have routinely practiced* pain management techniques for most of lower limb *orthopedic* surgery such as open reduction and internal fixation (ORIF), wiring of knee fractures, intramedullary nailing, Sequestrectomy, screw as well as plating femoral fractures [13,19]. Due to the availability of a peripheral nerve stimulator and insulated needle, those Single regional blocks are also practiced in the study area but the efficacy was not investigated.

In addition, Practice of regional anesthesia and analgesia is relatively low in Ethiopia [20,21]. Studies were also insufficient showing the efficacy of LPB versus 3IN1B in the setup as well as in Ethiopia, Therefore, this study mainly aimed to compare the analgesic efficacy of singe shot LPB versus 3IN1B for postoperative pain control of thigh orthopedic surgery at the University comprehensive specialized hospital.

## 2 Methodology

### 2.1 Study design, period and area

Institutional based prospective cohort study was conducted from October 10, 2020 to March 30, 2021 at the University comprehensive specialized hospital. Ethical issue was approved and obtained from college of medicine and health science ethical review committee. The article has been registered with unique identifying number (UIN) of researchregistry7288. The study has been reported in line with the STROCSS criteria [22]. Informed consent was taken from each study clients after disclosing the purpose of their involvement and not harmed by their participation.

### 2.2 Inclusion and exclusion criteria

Adult Patients with elective thigh orthopedic surgery operated under spinal anesthesia, post-operative LPB or 3IN1B, age between 18 and 60 years, ASA Ⅰ and ASA Ⅱ were included. While patients with loss of consciousness, psychiatric patients, unable to communicate and understand, patients who were sedated and given further analgesia in the intraoperative time, spinal anesthesia other than bupivacaine, multiple trauma, patients with chronic pain or chronic pain analgesia usage and hospital discharge before 24hr were also excluded in this study.

### 2.3 Sample size determination

In a study done regarding, LPB vs. 3IN1B with bupivacaine, the mean duration of analgesia (mean ± SD) was 20.6 ± 5.7 and 15.8 ± 6.4 respectively [23]. Assuming 1:1 ratio in the two groups with the power of 90% and level of significance α = 0.05, the sample size was calculated by the formula of;

N= (α +p) ^2^ (sd1^2^+sd2^2^) / (μ1-μ2) ^2^

N = 1.96 + 1.28)^2^(5.7^2+^6.42)/ (20.6-15.8) ^2^

N = 33.47 = 34

Assuming none response rate as 15%, final sample size for each 39.1 = 40.

Therefore, the total sample size was 80.

α Level of significance 0.05 which is 1.96,
P Power of 90% = 1.28
SD 1 Standard deviation of analgesia in LPB block group
SD2 Standard deviation of analgesia in 3IN1B block group
μ1 Mean for the first time analgesia request in LPB block group
**μ**2 Mean for first time analgesia request in 3IN1B group

### 2.4 Sampling technique

Non-probability convenient sampling was conducted to select participants in both groups. Odd or even numbering method was used to allocate the study participants in either group. We took a proportional number of participants in LPB and 3IN1B (40 in each group) until the required calculated sample size of 80 was fulfilled.

### 2.5 Study variables

Time to first analgesia request, the severity of postoperative pain with and without movement and total analgesia consumption were the outcome variables of the study. Whereas, sociodemographic variables (age, sex, weight, height, BMI), ASA status, type of surgery, duration of surgery, duration of sensory recovery after spinal anesthesia, postoperative analgesia, and volume of spinal anesthesia were independent variables in this study.

### 2.6 Data collection procedure

Before spinal anesthesia, standard monitoring such as pulse oximetry, electrocardiography (ECG) and noninvasive blood pressure (NIBP) were attached and spinal anesthesia was given with aseptic technique. After the operation, either LBP or 3in1B was done by the responsible anesthetist.

Regarding the technique of blocks, the clients were in *the lateral position* with the side to be blocked up and lumbar spine flexed during LPB. Landmarks was *the point* of intersection between a line joining the upper *border* of *the iliac crest* and posterior superior iliac spine (PSIS). At this point the non-insulated needle was inserted perpendicular to the skin. If bone is contacted (transverse process) withdraw and redirected caudally with maximum of 20 mm until sustained contraction of the femoral quadriceps muscle at 0.5 mA. After gaining this contraction, single shot 30 ml of 0.25% bupivacaine was administered. While, the 3IN1B was also performed using the land mark of 1.5 cm below the inguinal ligament and 1.5 cm lateral to the femoral artery after putting the patient supine position. The needle was inserted perpendicular to the skin and after obtaining sustained contraction of femoral quadriceps muscle at 0.5 mA, single shot 30 ml of 0.25% bupivacaine was given.

The clients were informed to ask pain killer while they started to feel pain at any time, so that the assigned nurses could manage. This time was recorded as the first request of analgesia. Intravenous tramadol and intramuscular Diclofenac were administered in both groups of patients feeling moderate to severe post-operative pain.

The time to first analgesic request in the first 24hr was the primary outcome while, the postoperative pain severity and the total analgesic consumption were the secondary outcomes. Severity of pain was assessed with numerical rating scale in which patients were asked to rate their pain on a scale of 0–10, in line with no pain, mild pain, moderate pain, and worst pain experienced.

The Pain was assessed by data collectors at postoperative 2 h, 4 h, 6 h, 8 h, 12 h, and 24 h in post-anesthesia care unit and orthopedic ward or any ward where they were admitted to.

The data were collected by two trained anesthetists with the questioner prepared in English version and translated into Amharic language (local as well as official language). The investigators of the study couldn't decide about the type of block given to the patient, but the responsible anesthetist can choose the type of nerve block according to her/his preference. The investigators stayed inside the operating room and observed the type of nerve block done by the responsible anesthetist and put specific codes on the patient chart. The type of nerve block was not stated clearly on the patient chart except the specific code. Therefore, the data collectors were blinded to the types of nerve block done for each patient.

### 2.7 Data processing and analysis

After completion of the data collection, entered into Epi-data software for checking and cleaning of errors. Then, transferred into SPSS (Statistical package of social science).

. Analysis was done by using SPSS version 20 statistical package. Shapiro–Wilk normality test was used to check the normality of the data. In this study, normally distributed data were analyzed using Student's independent *t*-test, and then the result was presented as mean ± SD (standard deviation). Whereas, non-normally distributed variables were analyzed by Mann–Whitney *U* test and the result was expressed as median with (interquartile range). The comparisons of categorical parameters were analyzed using the chi-square test and Fisher's exact test as required and expressed in numbers and percentage. Finally, P value < 0.05 was considered as statistically significant.

## 3 Result

### 3.1 Socio demographic characteristics

A total of 80 participants were enrolled in this study. Of those, 40 patients were receiving LPB while the other 40 patients were given 3IN1B. The mean age of the patients was (mean ± SD) 27. 40 ± 9. 45 years (age range 18–55). The demographic characteristics of the participants (weight, height, BMI, age, dose of spinal and ASA physical status) and surgical duration were comparable between the two groups. In this study, 77% of the 3IN1B group and 82% of the LPB were males (Table 1). In addition, 35% of the 3IN1B and 38% of the LPB groups were ASA Ⅰ as well as 65% of the 3IN1B and 62% of the LPB groups were ASA Ⅱ patients. The mean duration of sensory recovery of spinal anesthesia after the end of the procedure when assessing the contralateral leg was comparable between the groups.

**Table 1:** Sociodemographic characteristics of the study participants, (N = 80).

Character	LPB(n = 40)	3 in1B(=40)	p-value
Age(years)	27.27 ± 8.64	25.85 ± 7.55	0.41
Sex (Male/Female)	33/7	31/9	0.55
Height (cm)	172.88 ± 6.98	171.71 ± 5.9	0.45
Weight(kg)	64.64 ± 7.18	62.29 ± 9.53	0.25
BMI(kg/m^2^)	21.54 ± 1.94	21.02 ± 3.05	0.4
Spinal dose(ml)	3.17 ± 0.30	3.19 ± 0.32	0.82
Duration of surgery(hr)	2.76 ± 0.70	2.8 ± 0.88	0.84
Duration of SR of SA after operation	1.6 ± 0.8	1.75 ± 0.5	0.3

Abbreviation: LPB, lumbar plexus block; 3IN1B, three -in-one block; kg, kilogram; m^2,^ meter square; cm, centimeter; milliliter; hr, hour: SR, sensory recovery.

Regarding the hemodynamic status, there was a statistically significant difference in mean arterial pressure (MAP) at 2hr, 4hr, and 6hr (Table 2), but pulse rate (PR) was comparable between the two study groups (Table 3).

**Table 2:** Postoperative mean arterial pressure (mmHg) in the two groups after regional block, (N = 80).

Post-operative time	LPB(n = 40)	3IN1B(n = 40)	P-value
2 h	72.20 ± 13.17	80.56 ± 11.38	0.007
4 h	74.44 ± 10.30	80.46 ± 9.5	0.015
6 h	76.70 ± 8.84	83.41 ± 9.90	0.004
8 h	77.70 ± 7.34	83.12 ± 9.23	0.01
12 h	80.17 ± 8.35	83.20 ± 6.2	0.17
24 h	81.47 ± 7.88	81.06 ± 8.86	0.84

Abbreviation: LPB, lumbar plexus block; 3IN1B, three -in-one block.

**Table 3:** Postoperative pulse rate (beats per minute) in the two groups after regional block (N = 80).

Post-operative time	LPB(n = 40)	3IN1B(n = 40)	P-value
2 h	79.73 ± 11.45	80.53 ± 10.29	0.76
4 h	80.56 ± 11.22	81.47 ± 10.18	0.73
6 h	83.41 ± 9.65	83.26 ± 8.70	0.95
8 h	83.38 ± 6.7	84.18 ± 8.9	0.71
12 h	82 ± 9.52	85 ± 9.23	0.17
24 h	82.79 ± 9.42	85.23 ± 9.07	0.28

Abbreviation: LPB, lumbar plexus block; 3IN1B, three -in-one block.

Type of operation and type of block is seen in (Table 4).

**Table 4:** Showing the relationship between the type of operation and type of nerve block, (N = 80).

Type of operation	LPB(n = 40)	3 IN 1B(n = 40)
Anterograde IMN	12	10
Retrograde IMN	9	10
Plating	5	3
IMN correction	1	1
Femoral neck fixation	3	4
ORIF	8	10
Sequestrectomy	1	3

Abbreviation: ORIF, open reduction and internal fixation; MN, intramedullary nailing; LPB, lumbar plexus block; 3 in 1B, three in one block.

The mean and standard deviation for the 1st request of analgesia was 11. 55 ± 2. 82 in LPB group and 13. 35 ± 2. 60 in the 3IN1B group (p - value of 0.07) (Table 5). On the other hand, regarding postoperative analgesic consumption, the mean tramadol and Diclofenac consumption was not statistically significant.

**Table 5:** Time to 1st request of analgesia and postoperative total analgesia consumption (N = 80).

Character	LPB (n = 40)	3IN1B(n = 40)	p-value
1st analgesic request time(hr)	11.55 ± 2.82	13.35 ± 2.60	0.07
Total tramadol consumption(mg)	67.65 ± 27.20	70.59 ± 37.19	0.71
Total diclofenac consumption(mg)	63.23 ± 45.74	44.88 ± 34.72	0.07

Abbreviation: hr, hour; LPB, lumbar plexus block; 3IN1B, three -in-one block.

The study showed that there was no statistically significant difference in postoperative pain score between the two study groups in both at rest and with movement (Table 6, Table 7).

**Table 6:** *Postoperative NRS at rest over the first postoperative* 24hr*, (N = 80).*

Post-operative time	LPB(n = 40)	3 IN B(n = 40)	P-value
2 h	0(0–0)	0(0–0)	0.89
4 h	0(0–0)	0(0–1)	0.48
6 h	0(0–1)	0(0–1.25)	0.27
8 h	1(0–2)	1(0–2)	0.52
12 h	1(1–2.5)	1.5(1–3)	0.62
24 h	2(2–3)	2(1.75–3)	0.09

Abbreviation: LPB, lumbar plexus block; 3 IN 1B, three in one block; NRS, numerical rating scale.

**Table 7:** Postoperative NRS on movement (physiotherapy or active movement of the foot) over the first postoperative 24hr, (N = 80).

Post-operative time	LPB(n = 40)	3IN1B(n = 40)	P-value
2 h	0(0–0)	0(0–0.25)	0.52
4 h	0(0–2)	0(0–1.25)	0.90
6 h	0(0–3)	0.5(0–3)	0.42
8 h	1.5(0–3)	2(0–3)	0.63
12 h	3(3–4)	3(3–4.25)	0.31
24 h	3(2.75–4)	3(3–4)	0.57

Abbreviation: LPB, lumbar plexus block; 3IN1B, three -in-one block; NRS, numerical rating scale.

## 4 Discussion

In this prospective cohort study, both single shot LPB and 3IN1B were comparable in the mean time to 1st request of analgesia, postoperative pain score both at movement and at rest and total analgesic consumption for the first 24hr after thigh orthopedic surgery. The result of this study was comparable with different studies done previously [14,15,24]. This study was also supported by another study of continuous catheter-based LPB and 3IN1B. However, our finding was different with another study concluding that LPB is more effective than 3IN1B, regarding total analgesic consumption, it was higher in the 3IN1B group than LPB, but similar the mean time to the 1st request of analgesia [18]. The possible explanation might be the previous study combined sciatic block in both groups for intra-operation anesthesia and postoperative analgesia.

This finding was also different from the previous study concluding that femoral nerve block has no effect compared with LPB after total hip arthroplasty [25]. The reason might be the hip is innervated by the lumbosacral trunk and branches of the lumbar plexus before forming the femoral to form the femoral, lateral femoral, and obturator nerves. However, comparison of 3IN1B vs. IV morphine for pain management of fractured hip showed that significantly decreased total analgesia consumption and NRS in the 3IN1B group [26].

Study done by S. Ponnambalam Namasivayam and his colleagues regarding LPB versus 3IN1B for post orthopedic surgery, lower limb pain management stated that both blocks were similar analgesic efficacy. The mean 1st analgesia request time was 9.10 (±1.52) in 3IN1B and 9.90 (±1.21) in LPB [27], which was similar to this study finding.

Another study conducted by Mohammed Abid Ziyauddin Chauhan regarding LPB vs.3IN1B concluded that single shot lumbar plexus block and 3IN1B had effective postoperative analgesia for lower limb orthopedic surgery and the 1st request time was 12.56 ± 3.91 and 11.83 ± 3.84 [28] which was comparable to the current study.

In contrast to our findings, the study done by Imbelloni and his colleagues comparing psoas compartment block with inguinal paravascular block found that psoas compartment block was more effective regarding time to 1st analgesia request, total analgesia consumption and pain reduction quality [23]. The mean duration of the analgesia was 20.6 ± 5.7 h in the psoas compartment block and 15.8 ± 6.4 h in inguinal perivascular. This difference to our results might be that the previous investigators used 40 ml of 0.25% bupivacaine for both groups.

A study done by Christopher J et al. to compare spinal anesthesia, LPB, and general anesthesia for knee arthroscopy surgery stated that the overall resource utilization was significantly decreased including total analgesia consumption, inra-operation and postoperative patient condition, duration of stay in post-anesthesia care unit and satisfaction with pain management in patients who underwent spinal anesthesia and LPB [29].

The results of our study showed that there was a significant difference regarding the mean arterial pressure until 8hr post-regional block between LBP and 3IN1B. The reason might be LPB has a unilateral sympathetic block [19]. This was also supported by other evidence stating that 3IN1B was more effective with less complications as compared with LPB in patients who had undergone total knee replacement surgery [15]. A study done in Egypt on Quadratus *Lumborum* (QLB) vs. Fascia Iliac block (FIB) for postoperative pain management of hip arthroplasty supports the current study result, which stated that there was a significant drop of blood pressure in QLB compared to FIB in the post block time, but had comparable analgesic efficacy [30].

Taherzadeh and his colleagues compared 3IN1B vs. intravenous morphine for pain management with *a fractured femur* and there was significant pain relief and total morphine consumption in 3IN1B group compared with IV morphine [31].

Another study done regarding lower limb block with intrathecal bupivacaine with and without adjuvant, the result showed that addition of dexmedtomidine prolonged postoperative analgesia compared to bupivacaine with or without clonidine. However, compared with the lumbar plexus and three -in-one nerve blocks, single shot intrathecal bupivacaine with and without adjuvant may have a short time to 1st analgesia request [32]. Single shot caudal and lumbar plexus blocks have similar intraoperative and postoperative opioid requirements, furthermore, they have more or less comparative postoperative pain scores after pediatrics hip surgery [33].

## 5 Limitations of the study

The study is observational, Patients were not randomly allocated even though there were *homogeneous* comparable groups. Not assessed regarding, Pre-operative pain, pre-operation analgesic medication, small sample size, complications related to blocks and shorter duration of postoperative follow-up. There was also covid 19 during data collection period which might affect the quality of information provided by participants.

## 6 Conclusions and recommendations

The study showed that there was no statistically significant difference between LPB and 3IN1B in the time to 1st request of analgesia, postoperative pain score at each time point both at rest and movement and total analgesic consumption.

Therefore, according to the current study, we recommended to use either LPB or 3IN1B for effective pain control after thigh orthopedic surgery. We also suggest further studies with longer postoperative follow-up involving the above-mentioned limitations.

## Ethical approval

University of Gondar college of medicine and health science.

## Sources of funding for your research

University of Gondar college of medicine and health science.

## Author contribution

**Authors' contributions** this work was carried out in collaboration among all authors. **HA (Habtu Adane)** contributed to the conception and design of the study, acquired; analyzed and interpreted the data drafted and revised the manuscript. **AT (Abraham Tarekegn) and YA (Yonas Admasu)** participate in reviewing the design and methods of data collection, interpretation and preparation of the manuscript. All authors participate in preparation and critical review of the manuscripts. In addition, all authors read and approved the manuscript.

## Research registration unique identifying number (UIN)

Researchregistry7288

https://www.researchregistry.com/browse-the-registry#home/

## Guarantor

Habtu Adane Aytolign.

## Consent

Written informed consent was obtained from every study participant after a clear explanation of the purpose of their participation and not harmed by participants. Anyone who was not willing to participate in the study was informed that he or she would have the full right not to participate or stop at any time.

## Provenance and peer review

Not commissioned, externally peer-reviewed.

## Data availability

All data generated or analyzed during this study are included in this article and found on reasonable base.

## Declaration of competing interest

**The authors declare that there is no conflict of interest**.

## Abbreviations

ASA: American society of anesthesiologist
BMI: ody mass index
IV: Intravenous
3IN1B: Three in one femoral nerve block
LPB: Lumbar plexus block
NRS: Numerical rating scale
SPSS: Statistical package of social science
VS: Versus
